# Tunable multiple Fano resonance employing polarization-selective excitation of coupled surface-mode and nanoslit antenna resonance in plasmonic nanostructures

**DOI:** 10.1038/s41598-019-38708-2

**Published:** 2019-02-20

**Authors:** Jietao Liu, Zhi Liu, Haifeng Hu

**Affiliations:** 1 School of Physics and Optoelectronic Engineering, Xidian University, Xi’an, 710071 China; 2 State Key Laboratory on Integrated Optoelectronics, Institute of Semiconductors, Chinese Academy of Sciences, Beijing, 100083 China; 3 College of Information Science and Engineering, Northeastern University, Shenyang, 110819 China

## Abstract

Modeling and tailoring of multispectral Fano resonance in plasmonic system employing nanoslit-antenna array is demonstrated and investigated. Efficient control of the multiple Fano profile can be manipulated, where the overall spectral is achieved by the separate contributions from the fundamental subgroups plasmonic resonance eigenstates. A polarization-selective strategy on nano-antennas resonance is proposed to shed light on the efficient manipulation of the multiple Fano resonances. Theory prediction of TM_−1_ surface mode excited in the system and thorough dispersion analysis of the supported Bloch modes provides evidence for understanding the origin of the transmission spectra. Compact nanophotonics planar optical linear-polarizer in the proposed nanostructure is investigated and demonstrated, where flexible Fano resonance control over the profile, linewidth and spectral contrast is appealing for applications such as sensing, switches and multifunctional nanophotonics devices.

## Introduction

Fano resonance in plasmonic system has been widely investigated, which has attracted vast scientific interest for their abilities to steer electromagnetic fields by tailoring of the subwavelength features and varied plasmonic resonances states^1–10^. More recently, in response to emerging applications such as biosensing and nano-switch, increasing attention has shifted towards obtaining enhanced control and tunability over all aspects of the spectra of the Fano resonance in plasmonic system^9–17^. There have been researches showing dynamically manipulated Fano resonance from coupling of resonance unit in in-plane metasurface system and out-of-plane coupling system^18,19^. More generally, metasurface system would introduce versatility and flexibility to nano-optics hardware that would circumvent the need for a new device to be fabricated every time when specific new optical response is required. For complex plasmonic nanostructures, the coherent resonance arising from hybridization of plasmon modes exhibits rich resonance spectrum^20–26^. However, more general method and simpler designed structures to control flexibly all aspects of the Fano (featured by its linewidth, spectral contrast, peaks and asymmetric factors) is much desired for versatile and convenient manipulation of the optical response.

Due to the difficulty in tuning the dielectric constants or changing the geometry after fabrication, more convenient and efficient approach enabling dynamically all-optical and large tunability is eagerly expected^12–15^. Recent approaches are reported exploiting phase-change materials employing vanadium oxide and germanium-antimony-tellurium (GeSbTe/GST), which have refractive indices that changes in response to external electronic or thermal biasing. This external modulation approach is potentially efficient and convenient; the overall tuning on the resonance profile’s linewidth and strength is gradually manipulated. Though effective, these tuning mechanisms have limited bandwidth and operating wavelengths that are determined by the physical properties of the specific materials used.

Here, we show that polarization tunable multispectral-selective switchable Fano resonance can be realized in the planar plasmonic system with coupled nanoslits antenna periodic array. The tailoring of the nanoslits antenna resonance unit, the hybridization and combination of the slits resonance provide the multispectral resonance with notable tunable features, where gradually modulated “on” and “off” states can be obtained by switching incidence light polarization. Such a coupling strategy for tailored spectral resonance can serve as platform for programmable visible, infrared and terahertz plasmonic devices and metasurface for on-chip photonic manipulation and modulation applications. The dispersion of the system is theoretically analyzed and the excited Bloch surface mode are revealed. The polarization-selective transmission of the system exhibits quite well trends of the Malus Law, which implies potential and possibility for promising multifunctional compact optical elements applications.

## Results

### Structures and simulations

**Figure 1:**
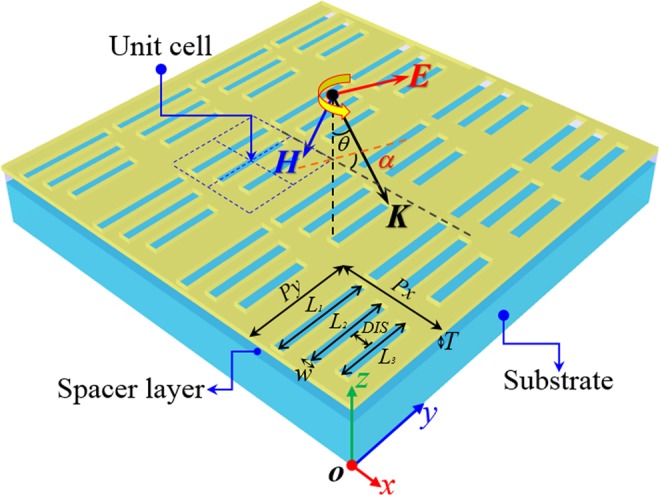
Schematic of the periodic nanoslits antenna array system. *E* and *K* stand for the electric component and the wave vector of the incident light, respectively. Unless any exception is noted, the array period are set as *Px* = 1000 nm, *Py* = 600 nm. The length of the slits: *L*_1_ = 400 nm, *L*_2_ = 300 nm, *L*_3_ = 200 nm. The width of the slits *w* = 100 nm, and the distance *DIS* = 150 nm. *θ* and *α* stand for the light incidence angle and the angle of polarization (AOP).

Figure 1 shows schematic of the system investigated, the system made up of hybrid-waveguide-plasmon nanostructure composed of thin gold film perforated with periodic array of nanoslits on a high-index dielectric slab waveguide spacer layer with a lower dielectric index material as the substrate. The scheme of the periodic array with gold nanostructures thickness h = 50 nm, slab waveguide layer thickness *T*, and periodicity along *x* direction *Px*, and *y* direction *Py*, is shown in the Fig. 1.

The incident linear-polarized light with the electric field polarization along x direction illuminates the system with an incident angle *θ* and angle of polarization *α*. The unit cell of the arrays consists of three parallel nanoslits antenna. The lengths of the slit antenna are labeled as *L*_1_, *L*_2_, *L*_3_, whereas the width w = 100 nm is equal and the distance between the slits *DIS* is 150 nm. Rigorous coupled wave analyses method is employed to model the optical properties of the structure. The dielectric constants of gold is fitted by Drude-Lorentz model^27,28^ and the refractive index of the substrate and the waveguide layer are set as *n*_*s*_ = 1.52 and n_*w g*_ = 2.1, respectively.

### Optical transmission response of the system

**Figure 2:**
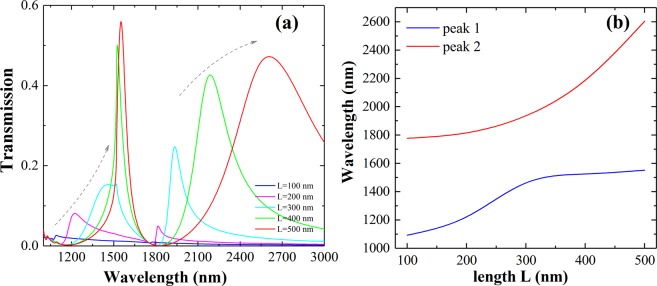
Calculated transmission spectra of the periodic array single nano-slit system. (**a**) The transmission of the single-slit array for varied slit arm lengths *L*. (**b**) Resonance peaks positions for different slit-lengths.

The peak transmission and the resonance conditions of a single rectangular aperture in a metal film are related to two main effects: Fabry-Perot resonance condition and cut-off condition^29,30^. To get deeper understanding of the system proposed, we investigate the system with single-nanoslit in the unit firstly. In Fig. 2(a), two global maxima in the transmission spectra are presented due to the excitation of two resonance modes, which are supported by the slits (same parameters as the system proposed)^29–31^. As predicted from theory and research on the single nanoslit situation, for the first resonance, the resonance linewidth decreases with the increases of the slit length, while the resonance is broaden for the second resonance. The overall resonance peak and the transmission are increased for increased slit length. Clear two main spikes in the transmission of the single-slit array system is attained.

The resonance peaks shift to long-wavelength for increased slit-length. For length value below and above 300 nm, the two branches for the resonance are separated apart gradually. For shorter slit length as 100 nm, the excited resonance is weak, and the transmission with two clearly recognizable spikes are vanishing (only two slight kinks in the transmission is seen). The results contribute to the design of the proposed system for multiple resonance and resonance manipulation.

**Figure 3:**
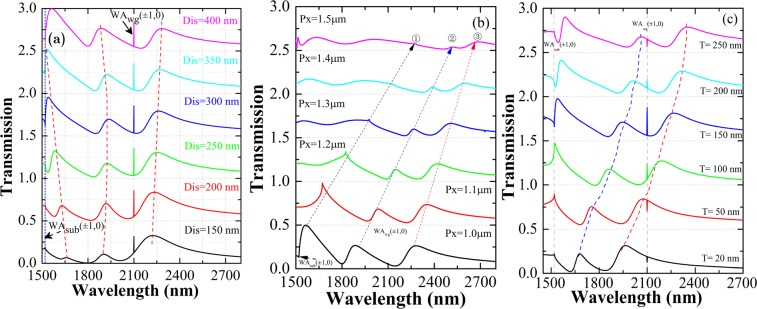
Transmission spectra showing modulated Fano lineshape. (**a**) The distance between adjacent slits *DIS* is varied from 150 nm to 400 nm. The period are fixed for *Px* = 1000 nm, *Py* = 600 nm, the waveguide layer thickness *T* = 150 nm. The red dashed lines depict a guide for view. The offset of the transmission is 0.5. Calculated transmission spectra of the system for varied period (**b**) and different spacer-layer thicknesses (**c**). The dashed lines with arrow have been added to guide the eye.

In the proposed system (see Fig. 1), owing to the nanoscale distance between the neighboring nanoslit antenna, the plasmonic resonance can exchange energy via their optical near-fields coupling. Therefore, the hybridization and combination of the coupled plasmonic modes is formed, where the superposition and convolution of the overall spectra is illustrated featured with multiple asymmetric Fano profile^20–22^. The coupled strength of the three slits in the unit cell can be controlled and the resulting Fano resonance spectra can be engineered accordingly (Fig. 3). One can see that, the three transmission peaks’ positions shift and the peaks value are changed as the distance between neighboring slits is varied. For smaller *DIS* value, stronger coupling of the individual resonance in slit is occurring; the third resonance in longer-wavelength zone shows stronger resonance strength and higher transmission than the first resonance (peak 1). While, for weak coupling situation with larger *DIS*, the first resonance is dominating, and the resonance at peak 2 is strengthening, the resonance at peak 3 shows slight shift to longer wavelength.

To gain insight into the physics of the origin of the spectral features, the dispersion for varied period *Px* is investigated. In Fig. 3(b), one can see that the multispectral Fano is maintained for varied periods, where the transmission is decreasing for the overall spectra. Nearly linear shifts of the resonance peaks with the lattice periods are observed. When light penetrates the metallic nanoslits, it excites Bloch modes inside the metallic-nanostructures layer, which are coupled directly (nonresonance process) or via SPPs (resonance process) to radiative diffraction orders^31^. The nonresonant excitation of quasiperiodic Bloch modes localized in the periodically distributed slits. The Wood Anomaly (WA) and the SPPs can be excited when the momentum-match condition is satisfied. The interference between the discrete process (resonant excitation of SPPs and Wood Anomalies) and the continuum (the nonresonant quasiperiodic Bloch modes) leads to Fano-type profile of the transmission spectrum.

The results in Fig. 3(c) shows the calculated zero-order transmission of the system proposed. Obvious spectral redshift with increased spacer layer thickness and array period is viewed. It is noted that for thinner spacer layer as 20 nm, the first peak in the Fano resonance spectra is vanishing, where an abrupt tuning in the transmission spectra is obtained. Sharp tuning in the first peak is shown and the peak position is fixed at 1540 nm for thickness *T* below 100 nm. For larger *T* values, thick waveguide layer that can support waveguide photonic mode is attributed to the abrupt tuning in the transmission, where the transmission peak is arisen. The peak 2 and peak 3 show obvious red-shift as the spacer layer thickness increases.

### Mode properties and polarization-tunability of the Fano resonance

**Figure 4:**
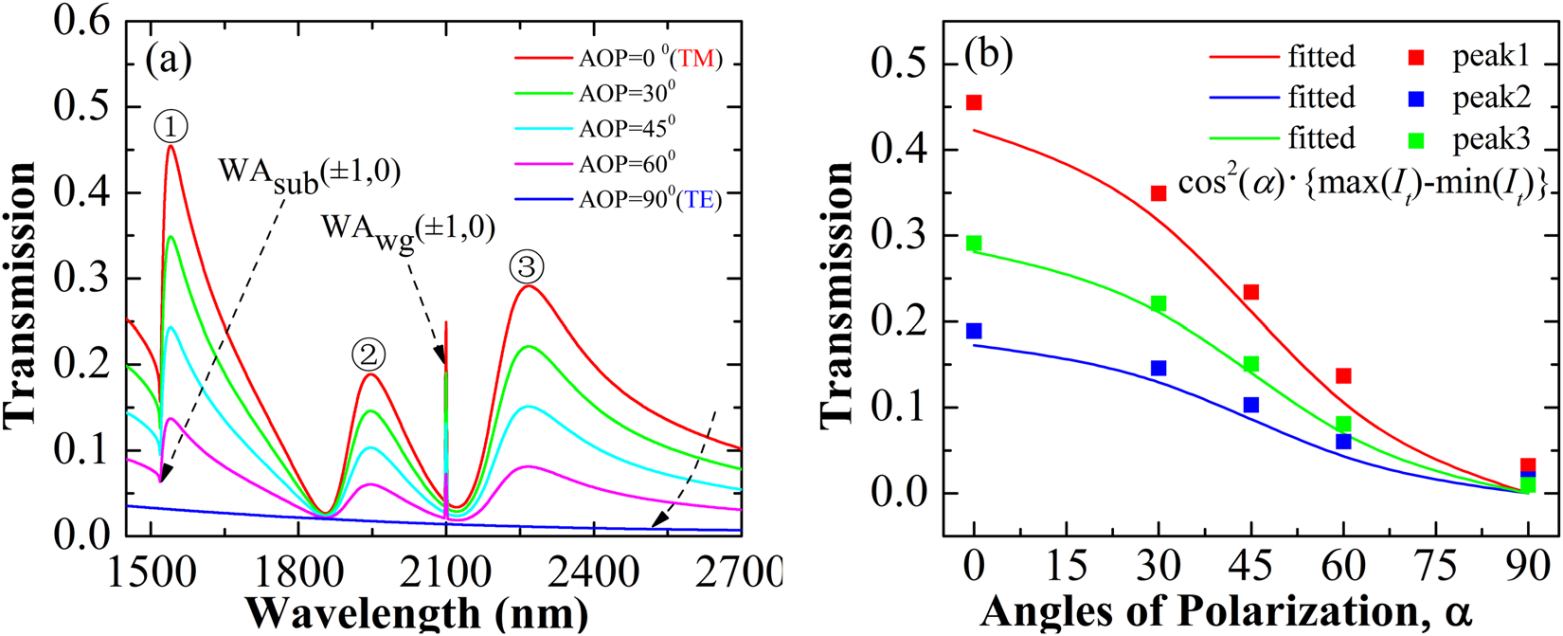
(**a**) The angle of incident polarization (AOP) is listed in the inset, where the Fano spectral can be gradually manipulated. (**b**) Transmission values for the three peaks. The scatters show the calculated transmission peaks values, and the solid-lines denote the theory-fitted results.

To further demonstrate the tailoring and manipulation of the overall spectra characteristic, the angle of incident polarization (AOP) is changed and the spectra evolution is investigated (Fig. 4). For varied polarization angles, the resonance peaks positions are fixed and the resonance strength is strongly related to the AOP. For polarization perpendicular to the slits’ long-arm, the resonance in the slits is excited and the transmission with Fano profile is seen; for parallel polarization incidence, the resonance in the slits cannot be excited, the transmission is suppressed and the Fano profile is faded away. The polarization dependence of transmission values at the resonance spikes are shown in Fig. 4(b), where the evolution for the transmission values is well described by Malus Law (modified cosines square relation with the maximum (max (*I*_*t*_)) and minimum (min (*I*_*t*_)) transmission values).

**Figure 5:**
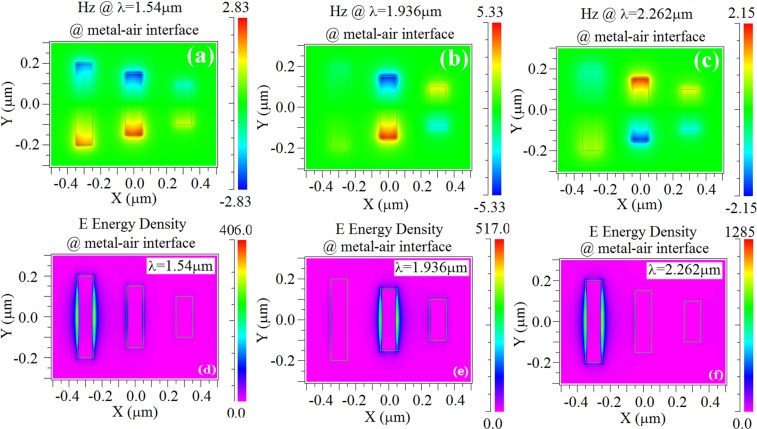
Calculated spatial field distribution of the magnetic field *Hz* component at the metal/air interface with normal incidence for *TM* polarization at the resonance wavelengths of 1.54 *μ*m (**a**), 1.936 *μ*m (**b**), and 2.262 *μ*m (**c**), corresponding to the transmission maxima in Fig. 3. (**d**–**f**) Calculated spatial electric field energy density distribution.

The transmission for varied AOP obeys the Malus law and changes according to the square of cosine (max(*I*_*t*_)-min (*I*_*t*_))*cos*^2^(*α*), which indicates that nearly pure polarized output beam passing out of the system can be effectively obtained. The system can serve as planar optical polarizer^32^, where the Fano resonance “on and off” state can be achieved. This opens new possibilities for all-optical controlled polarized-Fano resonance, thereby providing significant tool for developing novel nanophotonics applications such as sensors and detectors. To gain insight and further understanding of the spectral features, we investigate the spatial field distribution at resonance of the transmission peaks, and the results are shown in Fig. 5. The calculated spatial field distribution of the magnetic field *H*_*z*_ component with normal TM (Transverse Magnetic) polarization incidence corresponding to the maxima of the transmission in Fig. 3 (*Px* = 1000 nm, *Py* = 600 nm, *T* = 150 nm, and *DIS* = 150 nm,) is illustrated. For the first peak at 1.54 *μ*m, the dipole-like resonance in all the three slits is excited in phase, whereas nearly equal resonance strength in the two longer slits is found. For the second peak at 1.936 *μ*m (see Fig. 5(b)), strong dipole resonance in opposite phase is shown in the two neighboring shorter slits. For the third resonance peak at 2.262 *μ*m, the resonance feature of magnetic field distribution is similar to the resonance configuration for the second peak position (at 1.936 *μ*m), where the resonance in the two shorter slits is in-phase with each other and *π*-phase-shift with the longest slit resonance is represented.

To corroborate the above explanation, we have numerically calculated the electric field energy density on the upper interface of gold. Figure 5(d–f) shows the result for the TM-polarized case at three peaks wavelengths. The resonance shows dominating roles in different slits, for the cases of at wavelength of 1.936 *μ*m and 2.262 *μ*m. For the case of 1.54 *μ*m, the coupling of resonance in the neighboring slits is clearly seen from the energy distribution in the two longer slits.

## Discussion

**Figure 6:**
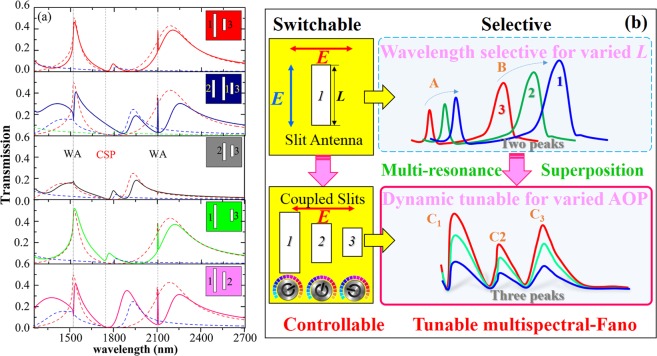
Tuning of resonance linewidth and spectral contrast of the resonance in different designed groups. (**a**) Calculated zero-order transmission spectra with designed nanoslits antenna (solid curve) and their subgroups (dashed-curves, and see result in Fig. 2(a)). The inset shows the group unit geometry. (**b**) Sketch of subgroup decomposing of the spectral profile and polarization-tunability of the system.

To dig into the multispectral formation and to elucidate the constitution influence, we vary the length of individual nanoslit and identify the role of the resonance coupling for the overall spectral. The Fano resonance spectra (see Fig. 6(a)) shows distinctive intensity modulation depending on the length of nanoslits, the whole spectra of the system can be tailored and the intensity modulation is related to the coupled strength of the adjacent individual nanoslit. The Fano profile is expressed as interference between discrete process and a continuum, the superposition of the multispectral Fano can be described by^2,31^:

1 $$T(\lambda )=C{(q{\rm{\Gamma }}/2+\lambda -{\lambda }_{res})}^{2}/((\lambda -{\lambda }_{res}{)}^{2}+{({\rm{\Gamma }}\mathrm{/2)}}^{2})$$

where q is an asymmetry factor, *λ*_*res*_ and Γ are the central wavelength and width of the resonance, and C is a parameter for normalizing the transmission. We intuitively illustrate the ability to program the overall spectra by tailoring the individual slit resonance and their coupling. The overall spectra for different groups (solid-lines in Fig. 6(a)) is tailored and the profiles can be well predicted by the superposition of its individual unit resonance spectra (dotted-lines). Different strengths of interference between the subgroups must be considered for a full understanding of the resulting spectral lineshape. The total spectrum of the system is the convolution of its separate subspectra, as well as with slight resonance shifts and linewidth variations. Understanding of the formation and the nature of the spectral response is significant for intuitive design and modification of the resonance profile using the decomposition idea. The hybridization and eigenstates superposition spectral design strategy opens up a rich pathway for the implementation and engineering of controlled optical properties for multichannel featured applications.

**Figure 7:**
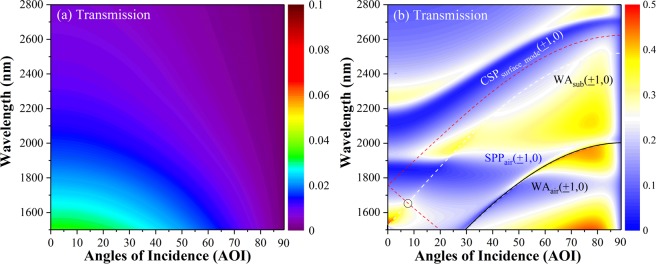
The dispersion of the system for TE incidence light (**a**) and TM polarization (**b**). The WA (white-dashed line) and the air-metal interface SPP Bloch-wave (black solid line) is denoted, which shows clearly the transmission energy distribution for varied AOI. The coupled surface-mode is analytically calculated (red-dashed-line).

In order to identify and distinguish different light coupling channels and regimes, the dispersion of the system is theoretically investigated. In Fig. 7(a), at normal incidence and transverse electric (TE) polarized incidence, gradually transmission decrease in a broad region is observed; for TM polarized incidence, however, the coupled Bloch modes of different orders occurs, leading to abrupt change in transmission (see Fig. 7(b)). The superimposed solid and white dashed lines are the calculated dispersion curves of air-metal interface SPPs Bloch-wave and WAs. The subscripts “sub” and “air” indicate the substrate and upper (air-metal) interfaces.

To consider the dispersion evolution and the role of periodic nanostructures, we propose a more rigorous description to treat the guided modes in the air/metal/waveguide/substrate multi-layered (four layer metal clad thin film optical waveguides) system. On the basis of Maxwell’s equations and appropriate boundary, the propagation constant for four-layer metal-clad coupled surface mode (CSP) of TM_−1_-surface-mode can be solved by the following equation^33^:

2 $$\tanh ({\gamma }_{1}T)=-\,({S}_{2}+{S}_{3}{\delta }_{3})/(1+{S}_{2}{S}_{3}{\delta }_{3})$$

here, S_*j*_ = (*ε*_1_/*ε*_3_)(*γ*_*j*_/*γ*_1_), (j = 2, 3, 4), *γ*_1_ = |${k}_{0}^{2}$ *ε*_1_ − *β*^2^|^1/2^, *γ*_*j*_ = (*β*^2^ − ${k}_{0}^{2}$ *ε*_*j*_)^1/2^, (j = 2, 3, 4), The parameters *δ*_3_ = ((S_4_ + S_3_) + (S_4_ − S_3_)exp(−2 *γ*_3_*t*))/((S_4_ + S_3_) − (S_4_ − S_3_)exp(−2*γ*_3_*t*)), *k*_0_ = *ω*/c, and the metal film thickness is *t*. In our design *ε*_1_, *ε*_2_, *ε*_3_ and *ε*_4_ are the dielectric constants of waveguide, substrate, metal, and air, respectively.

Under the Bragg coupling condition, the dispersion relation of the coupled Bloch mode can be obtained. The CSP mode for the four-layer-waveguide system is calculated and the dispersion curve (red-dashed-line) is shown in Fig. 7(b) (see Fig. 6(a), vertical line), which verifies that the coupled energy confines at the metal interface and transmission dip for varied AOI is quite well described (especially for non-normal incidence, see the black circle near AOI of 7°). This confirms the theory prediction and provides evidence for understanding the origin of the transmission.

The active control of the wavelength-selective switchable coupled multispectral Fano resonance can be feasibly tuned and designed by tailoring the unit geometry and by easily adjusting the angle of incidence.

## Conclusions

In summary, all-optically controllable multispectral Fano resonance was demonstrated in the proposed nanoslit array hybrid plasmon-waveguide nanostructures. Theory prediction of coupled TM_−1_ Bloch Surface Mode excited in the system and thorough theoretical dispersion analysis of the supported Bloch modes provides evidence for understanding the origin of the transmission. The wavelength-fixed polarization-selective multispectral Fano resonance and the switchable “on/off” state Fano transmission modulation is demonstrated, which is promising for tailoring the polarization behavior of multifunctional nanophotonics elements in many applications, including light polarization-modulation and ultrafast optical switching.
